# Discovery of Novel Lin28 Inhibitors to Suppress Cancer Cell Stemness

**DOI:** 10.3390/cancers14225687

**Published:** 2022-11-19

**Authors:** Mariia Radaeva, Chia-Hao Ho, Ning Xie, Sijie Zhang, Joseph Lee, Liangliang Liu, Nada Lallous, Artem Cherkasov, Xuesen Dong

**Affiliations:** The Vancouver Prostate Centre, Department of Urologic Sciences, University of British Columbia, 2660 Oak Street, Vancouver, BC V6H 3Z6, Canada

**Keywords:** Lin28 inhibitor, cancer stem cell, Let-7, zinc knuckle domain, RNA binding inhibitor

## Abstract

**Simple Summary:**

Cancer cells gaining stem cell gene signatures has been deemed as the root of cancers. Blocking the driver genes that control cancer stemness will lead to effective tumor suppression. Lin28 is a stemness driver gene that exerts its tumor-promoting activity through its RNA binding capability. Here, we analyzed the protein structure of Lin28 and discovered several chemicals that can block Lin28 RNA-binding activity. The identified Lin28 inhibitors strongly block the cancer cell stemness features, indicating that these chemicals may be further developed into clinically used drugs for cancer patients.

**Abstract:**

Lin28 is a pluripotency factor that regulates cancer cell stem-like phenotypes to promote cancer development and therapy-resistant tumor progression. It acts through its cold shock domain and zinc knuckle domain (ZKD) to interact with the Let-7 pre-microRNA and block Let-7 biosynthesis. Chemical inhibition of Lin28 from interacting with Let-7 presents a therapeutic strategy for cancer therapy. Herein, we present the computer-aided development of small molecules by in silico screening 18 million compounds from the ZINC20 library, followed by the biological validation of 163 predicted compounds to confirm 15 new Lin28 inhibitors. We report three lead compounds, Ln7, Ln15, and Ln115, that target the ZKD of both Lin28A and Lin28B isoforms and block Lin28 from binding Let-7. They restore Let-7 expression and suppress tumor oncogenes such as SOX2 in cancer cells and show strong inhibitory effects on cancer cell stem-like phenotypes. However, minimal impacts of these compounds were observed on Lin28-negative cells, confirming the on-target effects of these compounds. We conclude from this study the discovery of several new Lin28 inhibitors as promising candidate compounds that warrant further drug development into potential anticancer therapies.

## 1. Introduction

Lin28, Oct4, SOX2, and Nanog are the four core stem cell genes that can transform terminally differentiated fibroblasts into inducible pluripotent stem cells [1]. Lin28 is the only RNA-binding protein among these genes and had also been reported to regulate SOX2 and Oct4 expression in cancer cells [2,3]. During development, the expression of Lin28 appears as early as the two-cell stage [4] and it functions as the gatekeeper to control the transition between pluripotency and committed cell lineages [5]. Lin28 had been confirmed to be overexpressed in 15% of 527 primary human tumors and cancer cells from the lung, brain, ovary, breast, prostate, kidney, and other organs [6]. Numerous studies had also shown that elevated Lin28 expression is associated with high tumor metastasis and poor patient survival [6,7,8,9,10], and blocking Lin28 by either small molecule inhibitors or RNA silencing in various cancer lines and xenograft models suppresses cancer stemness phenotype and inhibits tumor progression [3,11,12,13]. Studies from our lab showed that the Lin28 regulates the cancer stem cell (CSC) gene network and promotes therapy-resistant neuroendocrine prostate cancer progression [3]. Lin28B knockout by CRISPR not only suppressed the CSC phenotypes of the NEPC cells but also prevented NEPC xenograft formation and tumor growth [3]. These findings rationalized that pharmacologically blocking Lin28 functions can be effective to control therapy-resistant tumors. 

Human cells have two Lin28 genes (Lin28A and Lin28B) that are not usually co-expressed in the same cell lineage [14]. Both isoforms shared high homology in their two RNA binding domains, the cold shock domain (CSD) and zinc knuckle domain (ZKD). The best-characterized function of Lin28 is to inhibit Let-7 miRNA biogenesis to exert its oncogenic activity. Crystallographic and biochemical studies demonstrated that the CSD and ZKD recognize the GNGAY (N = any base and Y = pyrimidine) and GGAG RNA motifs, respectively, in the terminal loop of Let-7 miRNA precursors [15]. In the cytoplasm, both Lin28 isoforms prevent the Dicer from accessing the GGAG motif, thereby inhibiting pre-Let-7 processing into mature Let-7 miRNA [16]. The Lin28B isoform can also be localized in the nucleus to block pri-Let-7 from being processed to pre-Let-7 by the Drosha and Dicer through similar mechanisms. Additionally, Lin28 can bind the GGAG motif of the stem-loops of mRNAs (e.g., Oct4) when folded into specific secondary structures [17,18], and further recruit RNA helicase A (RHA) to facilitate mRNA translation [19,20,21]. These studies demonstrate that Lin28 exerts its functions through its RNA binding to regulate let-7 biosynthesis and mRNA translation. 

Despite a consensus that RNA-binding proteins are not druggable, extensive efforts had been made to target Lin28 CSD or ZKD with small molecules (e.g., 1632, Li71, and KCB3602 et al.), using high-throughput screening techniques such as fluorescence polarization (FP) and fluorescence resonance energy transfer (FRET). Using FRET assays, compound 1632 was discovered to be capable of restoring Let-7 expression and suppressing cancer cell stemness [22]. The action of 1632 was shown to block the interactions between Lin28A and Let-7, however, the exact target site of 1632 within Lin28 has not yet been defined. The compound Li71 was discovered as a CSD binder and Lin28 inhibitor through FP assays [23]. Li71 presents high solubility and low cellular toxicity at high uM concentrations, but relatively low potency to inhibit Lin28 and suppress cancer stemness. These findings together indicated that Lin28 is a validated drug target and that blocking Lin28–RNA interactions will abolish the cancer cell stemness phenotype to achieve tumor cell suppression. However, further exploration of more candidate compounds and defining their mode of action (MOA) will improve the potency of Lin28 inhibition. 

Herein, we present the development of new Lin28 inhibitors that successfully block Lin28–RNA binding activity and suppress cancer cell stemness phenotype. These inhibitors were discovered by a computer-aided drug design (CADD) pipeline based on machine learning, facilitated molecule docking, and pharmacophore modeling. They target an interaction site between the Lin28 ZKD and the Let-7 GGAG motif and prevent Lin28 from inhibiting Let-7 synthesis. We confirmed fifteen compounds as Lin28 inhibitors and validated three lead compounds that can block the cancer cell stemness phenotype. We conclude with the discovery of the chemotypes of new Lin28 inhibitors that warrant further drug development as anticancer molecules.

## 2. Materials and Methods

### 2.1. CADD Methods

Binding site identification was performed in MOE [24] software (Chemical Computing Group, Montreal, Canada) using the SiteFinder tool. The protein structures used for docking and molecular dynamics were prepared using Protein Preparation Wizard from Schrödinger [25]. The preparation included hydrogen bond assignments (using PROPKA with pH of 7.0), minimization with an OPLS3e force field (with heavy atoms constrained to 0.30 Å RMSD), and addition of the missing side chains. Ligand preparation was performed using the Omega [26] toolkit (OpenEye Scientific, Santa Fe, CA, USA) and included canonization of SMILES, ligand tautomerization, and conformer generation. 

DeepDocking was performed with default parameters as described in Gentile et al., [27]. Redocking with GlideSP [25] was performed with default parameters. Docking grids were calculated with the grid generation software from Schrodinger. FRED docking was performed with default parameters. Distances between docking poses were generated using MOE [24] software. ADMET (absorption, distribution, metabolism, excretion, and toxicity) properties were calculated using SwissADME software [28].

### 2.2. Chemicals and Protein Purifications

ZINC compounds were purchased from Enamine (Kyiv, Ukraine), Life Chemicals Ltd. (Kyiv, Ukraine), or Princeton *Biomolecular* Research (Princeton, NJ, USA) with >90% purity. Upon receiving the compounds, the molecular masses were validated by LC/MS/MS. His-tagged human Lin28B ZKD (aa110-174), Lin28B CSD+ZKD (aa7-204), and Lin28A ZKD (aa121-187) were expressed in *Escherichia coli* BL21 cells and purified by immobilized metal ion affinity chromatography (IMAC) with nickel nitrilotriacetic acid (NiNTA) resin and size exclusion chromatography (s75 10/300 GL) as we have previously reported [29,30].

### 2.3. Fluorescence Polarization Assay

The fluorescein amidites (FAM)-tagged Let-7 probe with the sequence of UUAGGGCACGAGAUUUUGCCCACAAGGAGUU was purchased from Integrated DNA Technologies (IDT). The probe at the concentrations ranging from 1 nM to 50 uM was incubated with Lin28B (ZKD) or Lin28 (CSD+ZKD) to a final volume of 30 uL at room temperature for 20 min. To read the fluorescence, a TECAN 500 machine was used. Fluorescence polarization was calculated using the following equation:

$$P=\frac{F_{parrallel}-F_{perpendicular}}{F_{parrallel}+F_{perpendicular}}$$

where *P* is the fluorescence polarization reading, *F parallel* is the fluorescence intensity acquired from the parallel excitation plane, and *F perpendicular* is the fluorescence intensity acquired from the perpendicular direction of the excitation plane. The total fluorescence intensity is estimated using the following equation:

$$F=F_{parallel}+2F_{perpendicular}$$

All the raw FP signal data from the TECAN 500 FP results were analyzed using Prism 8.0 (GraphPad, San Diego, CA, USA); normalized curves were plotted and IC50 values were estimated. All FP data were repeated three to five times. 

### 2.4. Electrophoresis Mobility Shift Assay (EMSA)

EMSA followed the standard protocols we published with minor modifications [31,32,33]. An IRDye800CWN-labeled Let-7 RNA probe was purchased from IDT (Coralville, IA, USA). Lin28B or Lin28A proteins were incubated with 300 nM of probe in a standard 20 uL EMSA reaction in a reaction buffer containing 20 mM HEPES-NaOH, pH 7.4, 2 mM DTT, 3 mM MgCl_2_, 0.05% NP-40, protease inhibitors, and 0.1 ug poly[dI:dC]. After incubation for 30 min at 37 °C, reactions were loaded onto 4% non-denatured polyacrylamide gel in 0.5× TBE buffer. The RNA oligo bands were visualized by using the Li-Cor Odyssey 9120 Infrared Imaging System.

### 2.5. Bio-Layer Interferometry (BLI) Assays

BLI assays were performed as we previously reported [29,30] to quantify the direct and reversible interactions between small molecules and Lin28B ZKD by using OctetRED (ForteBio, Bohemia, NY, USA). Lin28B was thus expressed with His-tag and a biotinylated AviTag sequence (GLNDIFEAQKIEWHE) at its N-terminus in *Escherichia coli* BL21 cells. Purified Lin28B ZKD (50 ug/mL) was bound to the super-streptavidin sensors for 1 h at room temperature. Sensors were next blocked with biocytin, washed, and then dipped into wells containing increasing concentrations of the tested inhibitors in assay buffer (20 mM N-2-hydroxyethylpiperazine-N0-2-ethanesulfonic acid (HEPES), 150 mM NaCl, 500 uM tris(2-carboxyethyl) phosphine (TCEP), and 1% dimethylsulfoxide (DMSO)). Association for 200 s and dissociation in assay buffer for 100 s were recorded for each well. Data analysis was done using the OctetRED software (ForteBio, Bohemia, NY, USA).

### 2.6. Cell Line and Cell Maintenance and Other Routine Techniques

The DUNE cell line and DUNE cells with Lin28B knockout by CRISPR (DUNE (KO)) were reported in our previous studies [3,34]. IGROV1 cells were obtained from Sigma-Aldrich (St. Louis, MO, USA). DUNE and DUNE (KO) cells were cultured in Dulbecco’s Modified Eagle Medium with high Glucose/L-glutamine (DMEM; Hyclone, Logan, UT, USA) and 10% of fetal bovine serum (FBS; Gibico, Waltham, MA, USA). IGROV1 cells were cultured in Roswell Park Memorial Institute 1640 (RPMI-1640; Hyclone) medium with 10% FBS. All the cell lines were incubated in 5% CO_2_ at 37 °C and were tested for mycoplasma contamination monthly and cell line authentication was tested by short tandem repeat profiling by ATCC. 

There are several standard molecular techniques, including real-time qPCR, flow cytometry, cell proliferation, colony formation, and immunoblotting assays, that have been reported previously by our lab [3,29,30,35,36,37]. Primers and antibodies used in these techniques are reported in Tables S1 and S2.

## 3. Results

### 3.1. Identification of a Ligand Pocket for Inhibitors to Block Lin28B and RNA Interactions

**Figure 1:**
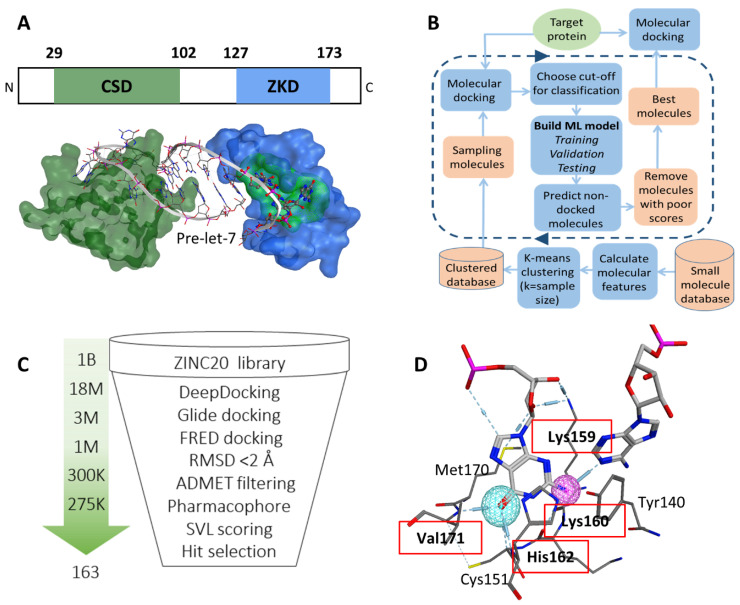
(**A**) The CSD (green) and ZKD (blue) domains interact with pre-Let-7 (white ribbon) in a bipartite manner (PDB: 5UDZ). The GGAG motif (red) and the surface of the proposed binding pocket (green) are marked. (**B**) A DeepDocking workflow is shown. First, for each molecule in the database molecular descriptors are calculated. These descriptors are then used to cluster all molecules. Second, molecules are sampled from clusters and docked into the target protein. Third, the derived docking scores and corresponding molecular descriptors are used to build machine learning models that predict the docking scores of undocked compounds. With each iteration, docking scores are predicted for a batch of molecules and the poorly scored molecules are removed. The number of iterations is user-defined for 5 rounds. The output is a list of molecules with predicted plausible docking scores. (**C**) The CADD pipeline is employed to predict 163 potential Lin28 ZKD inhibitors for biological testing. (**D**) Interaction between Lin28B ZKD and the two nucleotides (first guanine and the adenosine of the GGAG motif) from Let-7. The residues that form hydrogen bonds (in red dotted lines) are used as pharmacophore features and labeled by red boxes.

To identify a potential druggable site on the surface of Lin28, we examined both CSD and ZKD (Figure 1A). These domains recognize Let-7 in a bi-partite mode, where CSD binds the GNGAY motif and ZKD binds the GGAG motif of Let-7 [38]. First, we computationally searched for plausible drug-binding sites on the surface of both ZKD and CSD (PDB: 5UDZ) [39]. A cavity where the GGAG motif binds to the ZKD domain was scored at the top of potential druggable sites. We visually examined the cavity and found its width and depth are sufficient to accommodate a wide range of small molecules and provide the potential for strong ligand anchoring. We chose this ZKD GGAG binding site as the target expecting that small molecules docked into this pocket would displace the GGAG motif and block pre-Let-7 binding (Figure 1A). Second, we examined the literature and found that the GGAG motif is critical for pre-Let-7 processing as shown in deletion and substitution experiments [15]. Although both the CSD and ZKD are essential for pre-Let-7 degradation [15,40], the ZKD is the domain that directly recruits terminal uridylyltransferases (TUTases), TUT4, and TUT7, to oligouridylate the Let-7 RNA for degradation [39]. Additionally, ZKD recognition of the GGAG motif is required for TUTase recruitment, which is followed by the Dicer complex-mediated Let-7 degradation [16]. These studies indicated that disruption of Lin28 binding to GGAG would effectively block Lin28 activity in suppressing Let-7 biosynthesis. To summarize, both our computational observations and previous studies support that the identified docking pocket at the interface between Lin28 ZKD and the Let-7 GGAG motif is an ideal drug-binding site.

### 3.2. Virtual Screening to Identify Potential Inhibitors of Lin28B ZKD

A CADD campaign was initiated against the identified docking pocket on the surface of Lin28 ZKD. The crystal structure of Lin28 was downloaded from the Protein Data Bank (PDB: 5UDZ) [39]. Around the selected binding pocket there was no amino acid sequence difference between Lin28A and Lin28B forms; therefore, the published structure of Lin28 was considered a model suitable for both forms. We performed an initial virtual screening of 1 billion compounds from the Enamine library using the in-house developed DeepDoocking tool [27] coupled with Glide docking software [25]. Briefly, DeepDocking leverages the speed of machine learning docking score prediction to screen large libraries of compounds (Figure 1B). The whole ZINC20 database [41] was sampled to perform docking, and then the obtained docking scores were used to train models and predict the undocked compounds. In this way, we eliminated predicted poor binders, and the top 18 million compounds scored with DeepDocking were re-docked with Glide to retain 1 million compounds (Figure 1C). These compounds were then re-docked with FRED software (OEDocking by OpenEye) [42] and the spatial difference between the docking poses produced by the two software was computed. We removed compounds that have significantly different docking poses (RMSD >2 Å) retaining ~275,000 compounds that were then passed through ADMET filtering to eliminate potentially toxic compounds. Additionally, we screened the remaining library for molecules that would mimic the Lin28–GGAG interactions. We speculated that an active compound should form similar contacts with Lin28 residues as the native ligand, i.e., GGAG motif. To identify which residues contribute the most to the Lin28–GGAG binding, we ran a molecular dynamics simulation of the protein–RNA complex for 30 ns. We observed that residues Lys159, Lys160, His162, and Val171 form the most stable bonds with the ribonucleotides (Figure 1D). Therefore, these residues were then selected as pharmacophore features, which were used to filter the remaining 275,000 compounds yielding around 150,000. Additional scoring with MOE SVL scripts (e.g., number of hydrogen bonds, number of rings, logP) was performed to guide the expert selection of 163 compounds to be purchased for experimental validation. 

### 3.3. Biochemical Assays to Screen and Characterize CADD Predicted Compounds

**Figure 2:**
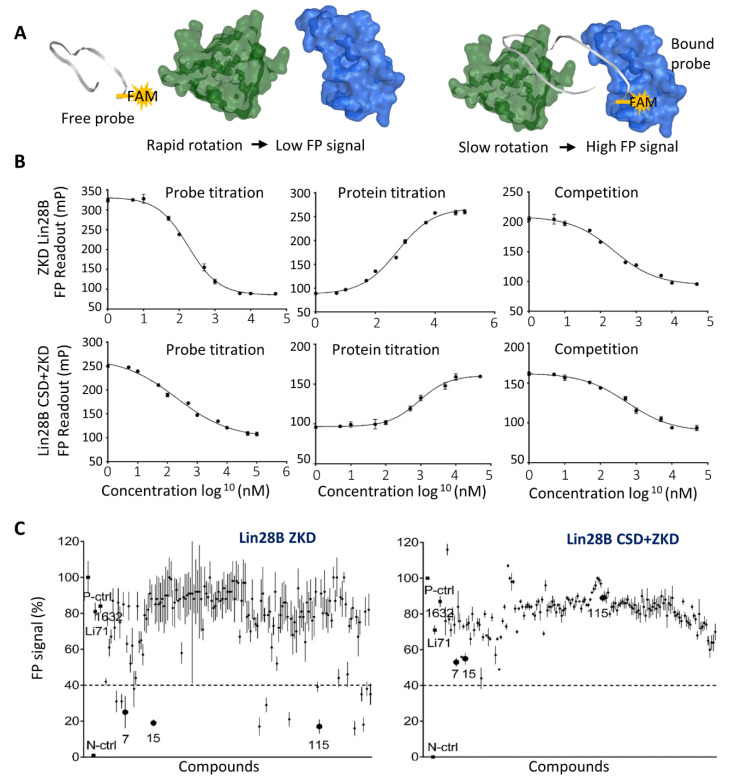
**Establishment of the Fluorescence Polarization Assay and Initial compound screening.** (**A**) Graphical representation of the FP assay principle. As the labeled probe floats freely in the solution, it rotates more rapidly, producing a low FP signal. When the labeled probe binds to proteins, it rotates slower and produces a high FP signal. (**B**) In FP assays, probe titration was performed by using 10 uM protein with 1 nM–100 uM FAM-labeled Let-7 probe (n = 9 replicates). Protein titration was performed by using 1 nM of FAM-labeled Let-7 probe with 1 nM–50 uM Lin28B ZKD protein (n = 9 replicates), and competition titration using 1 nM–50 µM non-labeled Let-7 probe with 10 uM of Lin28B ZKD protein with 1 nM FAM-labeled Let-7 probe (9 replicates). (**C**) FP assays were used to screen 163 compounds at the concentration of 20 uM using 1 nM of FAM-labeled Let-7 probe with 10 uM Lin28B ZKD protein.

To test the predicted compounds by CADD, we established an FP assay using both Lin28B ZKD and Lin28B CSD+ZKD to be incubated with a FAM-labeled Let-7 oligo containing the GGAG motif. To validate that the signals obtained from FP assays reflect the interaction between Lin28 and Let-7, we have tested: (1) 1 nM–50 uM FAM-labeled Let-7 probes with 10 uM Lin28 proteins and observed a dose-dependent reduction of FP signal; (2) 1 nM FAM labeled Let-7 probes with 1 nM–50 uM Lin28 proteins and observed a dose-dependent increase of FP signal; (3) 1 nM–50 uM doses of non-labeled Let-7 oligo to be incubated with 1 nM FAM probe and 10 uM Lin28 proteins, and observed a dose-dependent reduction of FP signal (Figure 2A,B). These results indicated that our established FP assays can be used to quantify the Lin28–Let-7 interactions and the effect of inhibitors on such interactions.

We then screened all 163 molecules predicted by the CADD pipeline by using the FP assays with 10 uM of Lin28B ZKD protein and 1 nM of FAM-labeled Let-7 probe. Compounds Li71 and 1632 were also included and served as positive controls. We used 60% inhibition of FP signals by 20 uM of the tested compounds as the cut-off and found 15 active compounds achieving a hit rate of 9% (15/163) (Figure 2C left). To confirm that all these compounds specifically target the ZKD, we have also repeated the FP assays using Lin28 CSD+ZKD, since CSD also has RNA binding capability. We found that no compounds achieved greater than 60% inhibition (Figure 2C right). Interestingly, the CSD inhibitor, Li71, showed ~30% inhibition of FP signals, while compounds Ln7, Ln15, and Ln115 resulted in about ~50, 50, and 20% inhibition in FP signals. These data suggested that the identified 15 ZKD inhibitors can be sufficient to block the interaction between ZKD and Let-7. Their inhibitory effects were relatively specific to ZKD, but not CSD, consistent with our rational drug by using CADD.

**Figure 3:**
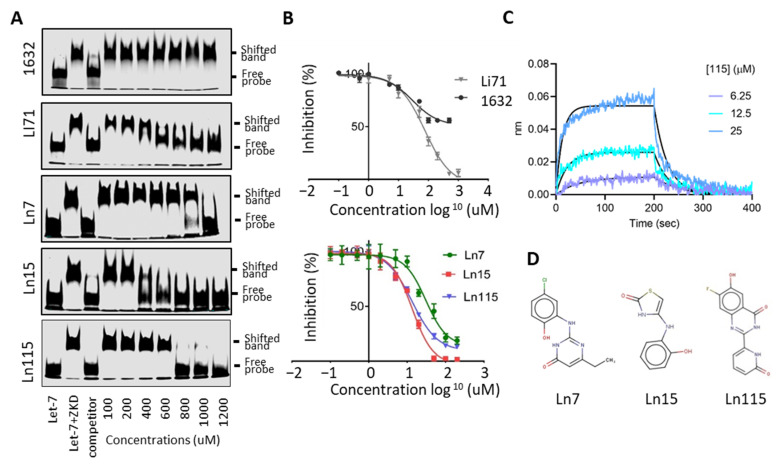
**Lin28 inhibitors block the interaction between Lin28B ZKD and Let-7.** (**A**) EMSA used 10 nM of IR800DyeCWN-labeled let-7 probe (lane 1), 0.5 uM Lin28B ZKD protein with 10 nM of labeled Let-7 probe (lane 2), or Lin28B ZKD, labeled Let-7 probe and 100× non-labelled competitor probe (lane 3). EMSA used Lin28B ZKD, labeled Let-7 probe, and 100–1200 uM of indicated compounds (lanes 4–10). (**B**) FP assays were performed by using 10 uM of Lin28B ZKD and 1 nM of FAM-labeled Let-7 probe and 1 nM–1000 uM of compounds. (**C**) Bio-layer interferometry (BLI) assays were performed using 0–25 uM of Ln115 with Avi-tagged Lin28B ZKD protein. (**D**) Chemical structures of the three lead compounds were shown.

To further prove that our identified compounds disrupt the interaction between Lin28B ZKD and Let-7, we performed electrophoresis mobility shift assays (EMSA) using concentrations ranging from 0 to 1200 uM of the studied molecules (Figure 3A). We observed robust inhibition by Li71 and not by 1632 of the complex between ZKD and Let-7 M. While Ln7 only inhibited ZKD–Let-7 interaction at the concentration > 1000 uM, Ln15 effectively disrupted the ZKD–Let-7 complex at 400 uM, and Ln115 showed the strongest efficacy that blocked ZKD–Let-7 interaction at 800 uM. Despite detecting a dose-dependent inhibition of ZKD–Let-7 interactions by the remaining 12 molecules by FP assays (Figure S1), we were not able to show this disruption by EMSA, most likely due to the lower sensitivity of EMSA. Therefore, we only focused on characterizing the compounds Ln7, Ln15, and Ln115 in the following assays.

We have titrated all these three compounds in FP assays to compare their potency in disrupting ZKD and Let-7 interactions. Compounds Ln7, Ln15, and Ln115 exhibited IC50s at about 45 uM, 9 uM, and 21 uM, respectively (Figure 3B), while the positive control Li71 had an IC50 of 55 uM, and compound 1632 can only achieve a maximum of 50% of inhibition. In summary, using two independent techniques we confirmed three lead compounds as Lin28B inhibitors.

FP and EMSA showed that three lead compounds disrupt Lin28–Let7 interactions. To validate that these molecules indeed directly bind to the ZKD domain of Lin28 in a reversible and dose-dependent manner, we used the biolayer interferometry (BLI) assay. Biotinylated Avi-tagged Lin28 ZKD was immobilized onto streptavidin biosensors. The sensor plates were then dipped in successive wells containing increasing concentrations of Ln7, Ln 15, or Ln115. BLI curves showed that Ln115 binds to ZKD in with an estimated KD of 60 ± 4 uM (Figure 3C), affirming a non-covalent binding of Ln115 that is consistent with our in silico simulation studies (Figure 1D). Despite several optimization trials, Ln15 showed non-specific binding to the sensors even in the absence of Lin28 ZKD protein, and could not generate reliable BLI signals. Additionally, Ln7 did not show any binding to Lin28 ZKD in the experimental conditions of the BLI assay (up to 100 µM), which suggests a weak binding affinity similar to what we have found in EMSA (Figure 3A). Nevertheless, even though the sensitivity of these three techniques (FP, EMSA, and BLI) are different our results together support that Ln7, Ln15 and Ln115 are Lin28 ZKD binders and inhibitors that prevent ZKD from interacting with Let-7 RNA. The chemical structures of these three compounds are shown in Figure 3D.

### 3.4. Lin28 Inhibitors Block Lin28 Functions in Cancer Cells

**Figure 4:**
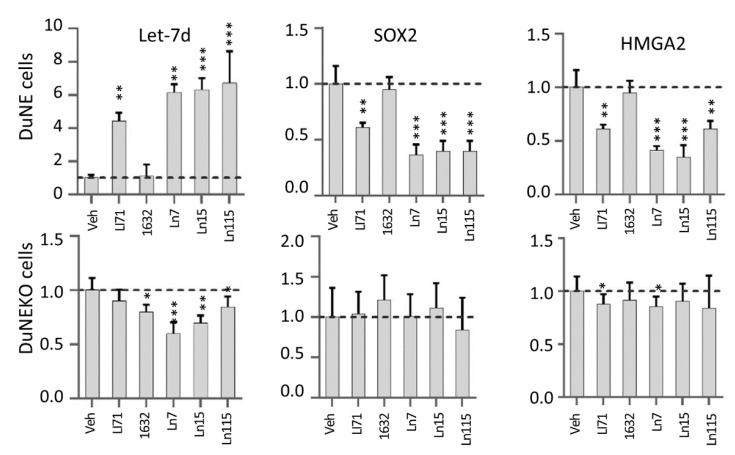
**Lin28 inhibitors restore Let-7d expression and suppress SOX2 and HMGA2 genes.** DUNE (**top**) and DUNE (KO) (**bottom**) cells were treated with 20 uM of the indicated compounds for 48 h. Real-time qPCR measured RNA levels of Let-7d, SOX2, and HMGA2. Three biological replicates were performed and all results are presented as mean +/− SD. *p*-value ≤ 0.05—(*); ≤0.01—(**); ≤0.001—(***).

Furthermore, we have tested if the lead compounds block Lin28 activities in cells and restore Let-7 miRNA expression (Figure 4). Previously, we have reported that Lin28B is highly expressed in the neuroendocrine prostate cancer cell line DUNE and that the major target of Lin28B in DUNE cells is Let-7d microRNA [3]. We have also reported that Lin28B upregulated the expression of the transcription factor HMGA2, which in turn stimulated SOX2 ^3^. When DUNE cells were treated with 20 uM of Ln7, Ln15, and Ln115 as well as control Lin28 inhibitors, Li71 and compound 1632, we observed that all three lead compounds and Li71 induced the expression of Let-7d levels by approximately six- to eight-fold. However, at the concentration of 20 uM, compound 1632 did not show any impacts on Let-7d expression. Consistently, HMGA2 and SOX2 mRNA levels were strongly inhibited by Ln7, Ln15, Ln115, and Li71 but not by compound 1632. SOX2 and HMGA2 protein levels were also reduced by our Lin28 inhibitors (Figure S2). These results indicated that our identified Lin28 inhibitors can block Lin28 activities resulting in the upregulation of Let-7 expression and downregulation of CSC oncogenes such as SOX2 in cancer cells.

To further proved that the suppressive effects of the Lin28 inhibitors were through blocking the Lin28B protein, we have repeated our real-time RT-PCR studies using the DUNE (KO) cells where the Lin28B gene was destroyed by the CRISPR technique. We showed that our identified Lin28 inhibitors have either marginal or no impacts on Let-7d, HMGA2, and SOX2 expressions, highlighting the on-target effects of these inhibitors in cancer cells. 

### 3.5. Lin28 Inhibitors Block Both Lin28A and Lin28B Isoforms

**Figure 5:**
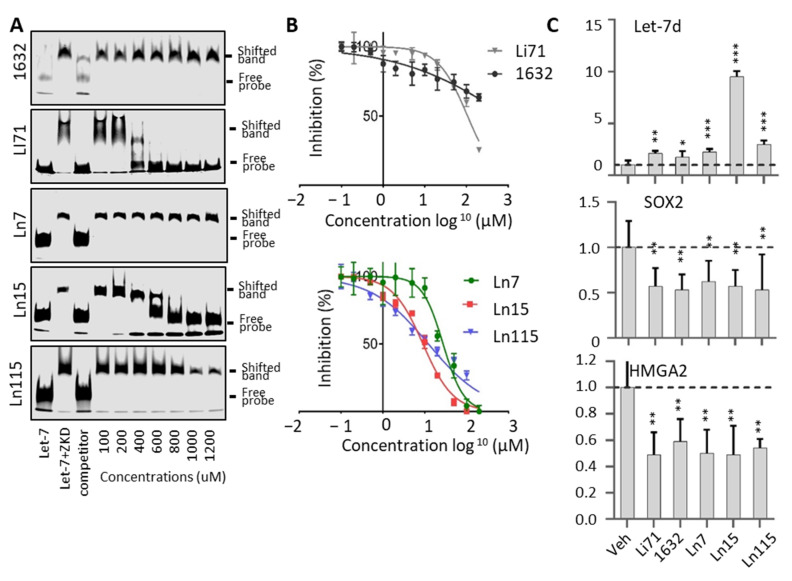
**Lin28 inhibitors block the Lin28A isoform.** (**A**) EMSA used 10 nM of IR800DyeCWN-labeled Let-7 probe (lane 1), 0.5 uM Lin28A ZKD protein with 10 nM of labeled Let-7 probe (lane 2), or Lin28A ZKD, labeled Let-7 probe and 100× non-labeled competitor probe (lane 3). EMSA used Lin28A ZKD, labeled Let-7 probe, and 100–1200 uM of indicated compounds (lanes 4–10). (**B**) FP assays were performed by using 10 uM of Lin28A ZKD, 1 nM of FAM-labeled Let-7 probe, and 1 nM–200 uM of compounds. (**C**) Lin28A-positive IGROV1 cells were treated with 20 uM of the indicated compound for 48 h. Real-time qPCR measured RNA levels of Let-7d, SOX2, and HMGA2. Three biological replicates were performed and all results are presented as mean +/− SD. *p*-value ≤ 0.05—(*); ≤0.01—(**); ≤0.001—(***).

Since Lin28A and Lin28B have a minimal amino acid difference around the docking pocket of our inhibitors, we speculate that our identified Lin28 inhibitors will block both Lin28 isoforms, thereby having much broader applications to treat various types of tumors driven by Lin28 signaling. We have repeated FP assays using Lin28A ZKD and observed that Ln7, Ln15, and Ln115 induced a dose-dependent suppression of FP signals with IC50s of 27.4, 9.1, and 12.2 uM, respectively (Figure 5A). In contrast, mild inhibitions of Li71 and compound 1632 were observed. We have also performed EMSA assays and found that Li71, Ln 15, and Ln115 at concentrations of ~100–400 uM inhibited the formation of the Lin28A ZKD–Let-7 complex, while compounds 1632 and Ln7 did not even at a concentration as high as 1200 uM (Figure 5B). To confirm that our Lin28 inhibitors can block Lin28A activity in cancer cells, we have used the IGROV1 cell model that expresses Lin28A but not Lin28B protein [43]. We observed that all three lead compounds as well as Li71 and compound 1632 upregulated Let-7 microRNA levels (Figure 5C). Ln15 even induced a 10-fold upregulation of Let-7. In addition, all compounds suppressed the expression of SOX2 and HMGA2 mRNA levels. Collectively, our data indicate that the newly discovered Lin28 inhibitors can block the activity of both Lin28 isoforms.

### 3.6. Lin28 Inhibitors Suppress Cancer Cell Stemness

**Figure 6:**
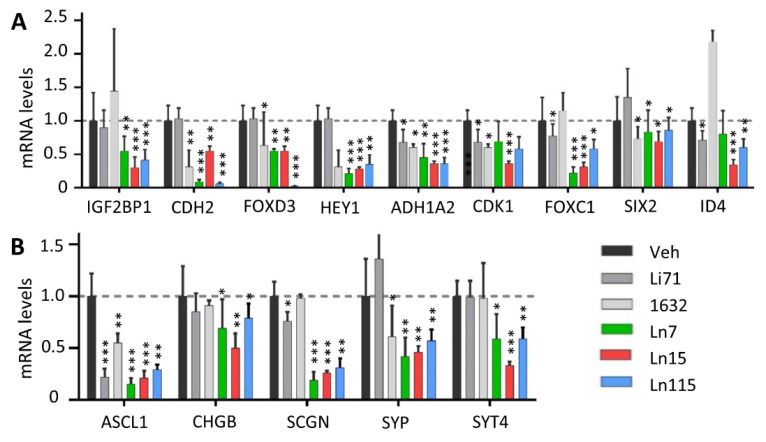
**Lin28 inhibitors block the expression of NE and CSC biomarkers.** DUNE cells were treated with 20 uM of the indicated compound for 48 h. Real-time qPCR measured RNA levels of neuroendocrine (**A**) and CSC (**B**) biomarkers. Three biological replicates were performed and all results are presented as mean +/− SD. *p*-value ≤ 0.05—(*); ≤0.01—(**); ≤0.001—(***).

**Figure 7:**
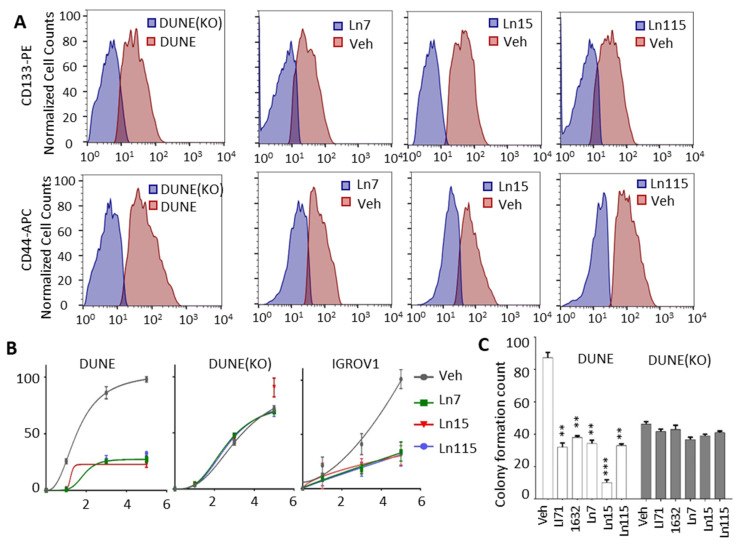
**Lin28 inhibitors block CSC phenotypes of cancer cells.** (**A**) FACS analysis using CD44 and CD133 antibodies detected CSC cell populations from DUNE or DUNE (KO) cells treated with either vehicle or 20 uM of Ln7, Ln15, and Ln115 for 48 h. (**B**) DUNE, DUNE (KO), and IGROV1 cell proliferation were measured in the presence of 20 uM of Ln7, Ln15, and Ln115. (**C**) Colony formation assays (colonies >50 uM were counted) measured the suppressive effects of Ln7, Ln15, and Ln115 at the concentration of 20 uM. *p*-value ≤ 0.01—(**); ≤0.001—(***).

The key roles of Lin28 in regulating cancer cell stemness and promoting therapy-resistant tumor progression had been demonstrated in many types of tumors [3,11,12,13]. We have shown that Lin28B drives cancer stemness phenotypes and promotes cancer cell lineage switch to a neuroendocrine lineage. We showed that Lin28B gene disruption inhibited cancer cell stemness and suppressed NEPC tumor progression. Therefore, we set out to test whether our identified compounds inhibit cancer cell stemness using our DUNE cell model. Four techniques were used to assess the capability of our Lin28 inhibitors to suppress the CSC phenotype. First, we measured the expression of a panel of CSC markers including IGF2BP1, CDH2, FOXD3, HEY1, ALDH1A2, CDK6, FOXC1, SIX2, and ID4 in DUNE cells by real-time qPCR. We confirmed the reductions of these CSC markers by Ln7, Ln15, and Ln115 (Figure 6A). Notably, Ln115 remarkably suppressed the expression of CDH2 and FOXD3 by up to 90%. In contrast, Li71 and 1632 showed variable mild inhibitory effects or no inhibition to several CSC markers. Furthermore, we also demonstrated that Ln7, Ln15, and Ln115 blocked the expression of several neuroendocrine markers including ASCL1, CHGB, SCGN, SYP, and STT4 (Figure 6B). Ln15 and Ln115 suppressed not only the mRNA expression but also protein expression of SYP (Figure S3). Second, we performed FACS to measure CSC biomarkers of CD133 and CD44 using DUNE cells treated with Lin28 inhibitors. We observed significant reductions of CD44- and CD133-positive cell populations in the presence of Ln7, Ln15, and Ln115. These changes were consistent with those from DNNE cells with Lin28B gene knockout (Figure 7A and Table S3). Third, we measured cell proliferation of DUNE (Lin28B positive) and IGROV1 (Lin28A-positive) cells. All three lead compounds resulted in up to 80% inhibition of cell growth. In contrast, no impacts were observed in DUNE (KO) cells, further validating that the inhibitory effects of our lead compounds act through targeting Lin28 proteins (Figure 7B). Fourth, we performed colony formation assays to evaluate the suppressive activities of Lin28 inhibitors to self-renewal and tumorigenesis capability of DUNE cells. We found that not only Ln7, Ln15, and Ln115 but also Li71 and compound 1632 strongly suppressed the formation of cancer cell colonies (Figure 7C and Figure S4). Ln15 showed ~90% suppressive effects. However, no inhibitory effects were observed using Lin28B knockout DUNE cells. Together, these studies confirmed that Ln7, Ln15, and Ln115 block cancer cell stemness by targeting Lin28 proteins. 

## 4. Discussion

We present a series of small molecule inhibitors that target Lin28 ZKD to disrupt its RNA binding activity resulting in the prevention of Let-7 microRNA degradation. These molecules block the CSC gene network and strongly suppress the clonogenic and self-renewal abilities of Lin28 positive cancer cells. The expression of Lin28 targeted SOX2, and HMGA2 oncogenes were also suppressed by these inhibitors, supporting that these compounds can block the pluripotency of tumor cells and suppress treatment-resistant tumor progression [44,45,46]. These results highlight the feasibility of Lin28 target therapy to abolish the CSC phenotype and suppress cancer recurrence, metastasis, and treatment resistance [47]. Since a rapidly growing body of research highlights the importance of Lin28 in various types of cancer [48], we propose that our lead inhibitors could be used as a starting point for the development of Lin28 target therapies. 

There was a notion that RNA-binding proteins are notoriously difficult to target with small molecules due to the lack of defined binding sites and generally disordered structures [49]. We dispute this conception and show that targeting an RNA-binding protein is feasible and highly translatable to modulate cellular phenotypes. Importantly, the conceived limitations were tackled with the use of computer-aided drug discovery technologies. Our computational analysis of the Lin28 surface allows the identification of druggable docking sites for small molecule binding. Additionally, the application of the novel machine learning-based tool DeepDocking enabled us to screen an ultra-large library of compounds. The coverage of such a broad chemical space ensured that the library of experimentally tested compounds is as focused as possible and provided a high chance of finding an effective inhibitor. Indeed, the hit rate of the initial FP assay was relatively as high as 9%, compared to high-throughput (HT) screenings of non-focused libraries such as HT FP screens on Lin28 protein, yielding 0.01% (14/16,000) [22], 0.05% (53/101,017) [23] and 2.3% (64/2,768) [50] hit rates. Most importantly, our inhibitors show superior activity compared to hits from the abovementioned screens (1632 and Li71, respectively). They showed minimal cytotoxicity to cells by measuring lactate dehydrogenase (LDH) release from cancer cells to culture media (Figure S5), and had no/low impacts to the expression of Let-7, SOX2 and HMGA2 in DUNE (KO) cells (Figure 4). The cell proliferation and colony formation of DUNE (KO) were also not affected by these Lin28 inhibitors (Figure 7). These results highlight low off-target effect of these inhibitors to low-Lin28 and Lin28-negative cells. Thus, this study further supports our previous conclusion that structure-based drug discovery enables the targeting of proteins conventionally considered to be undruggable [51].

## 5. Conclusions

In conclusion, we have discovered and characterized several new Lin28 inhibitors that have the potential to be developed into anticancer therapy for multiple types of cancers that are under the control of Lin28-regulated CSC gene networks.

## 6. Patents

Patents on Lin28 inhibitors are under consideration. Lin28 inhibitors can be made available to researchers after standard Material Transfer Agreement (MTA) implementation with the University of British Columbia.

## Data Availability

The data presented in this study are available in this article and the Supplementary Materials.

## Supplementary Materials

The following supporting information can be downloaded at: https://www.mdpi.com/article/10.3390/cancers14225687/s1, Figure S1: Lin28b ZKD titration with 11 selected chemicals; Figure S2: SOX2 and HMGA2 immunoblotting in the presence of three lead compounds; Figure S3: CD44, CD133 immunoblotting in the presence of three lead compounds. Figure S4: Colony formation assay. Figure S5: Cytotoxicity to cells by measuring lactate dehydrogenase (LDH) release. Table S1: Real time-qPCR Primer Information. Table S2: Primary Antibodies List. Table S3: CD133 and CD44 positive cell populations in the FACS studies in Figure 7 were listed.
