# Supplementary material for: Discovery of Novel Lin28 Inhibitors to Suppress Cancer Cell Stemness
Source: Cancers (Basel). 2022 Nov 19;14(22):5687. doi: 10.3390/cancers14225687 (PMC9688808; doi:10.3390/cancers14225687)
Supplement: Supplementary file 1 [file cancers-14-05687-s001.zip › cancers-1948087-supplementary.pdf]

Supplementary Information

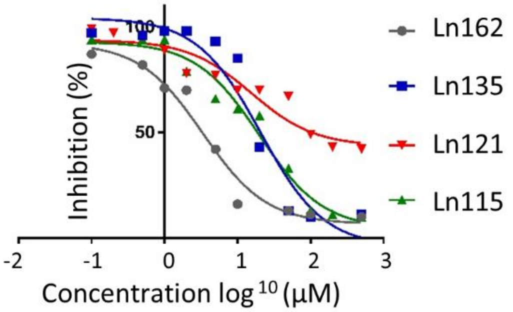

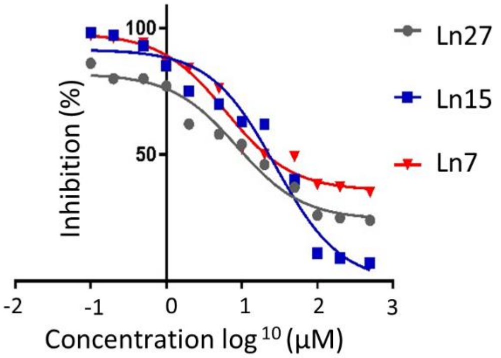

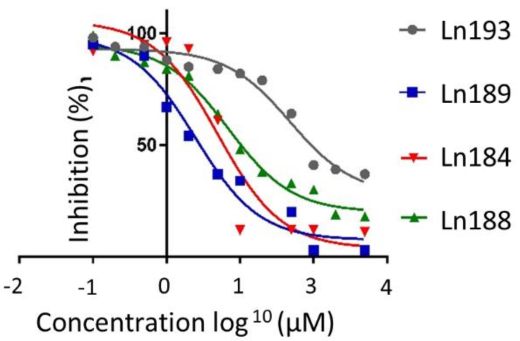

Figure S1. Lin28b ZKD protein was incubated with the indicated compounds at the concentration of 0, 0.1, 0.2, 0.5, 1, 2, 5, 10, 20, 50, 100, 200, or 500uM. FP signals were measured and plotted relative to that from vehicle.

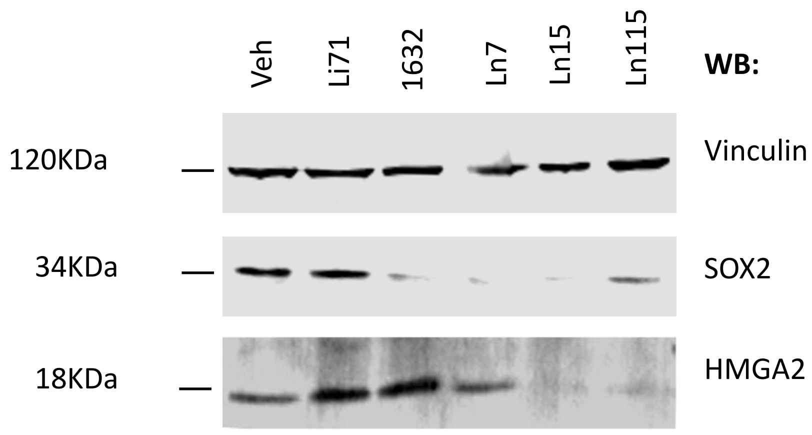

Figure S2. DUNE cells were treated with 20uM of indicated compounds for 24 hours. Protein lyses were collected and immunoblotted with Vinculin, SOX2, and HMGA2 antibodies.

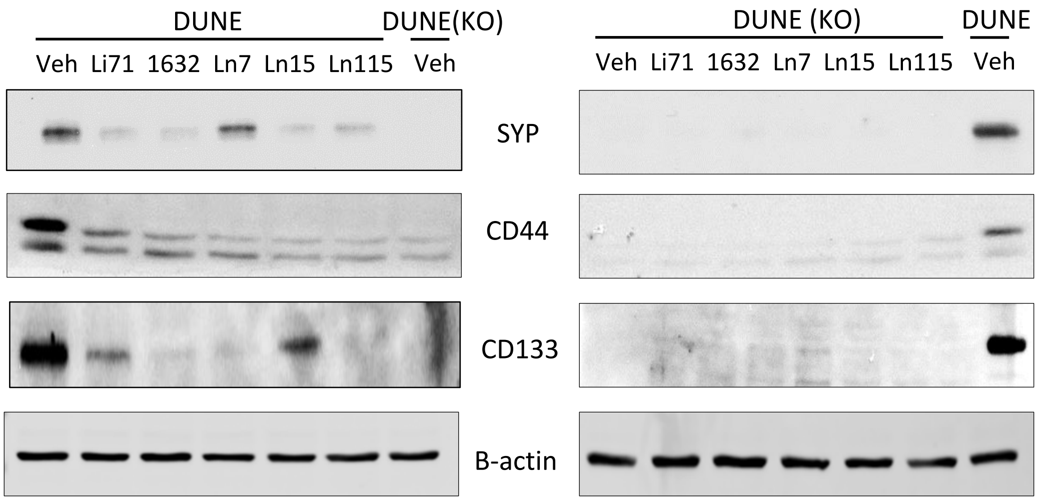

Figure S3. DUNE or DUNE(KO) cells were treated with 20uM of indicated compounds for 24 hours. Protein lyses were collected and immunoblotted with Lin28b, SYP, CD44, and CD133 antibodies. Beta-actin was used as the loading control.

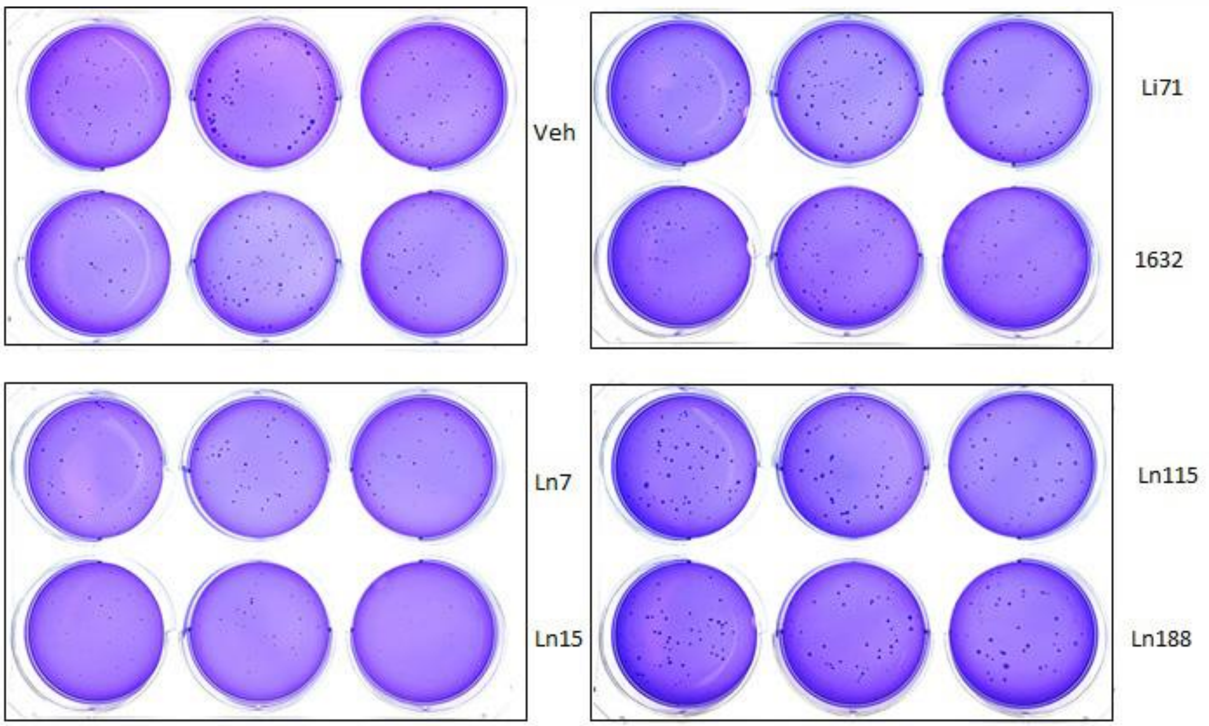

(A)

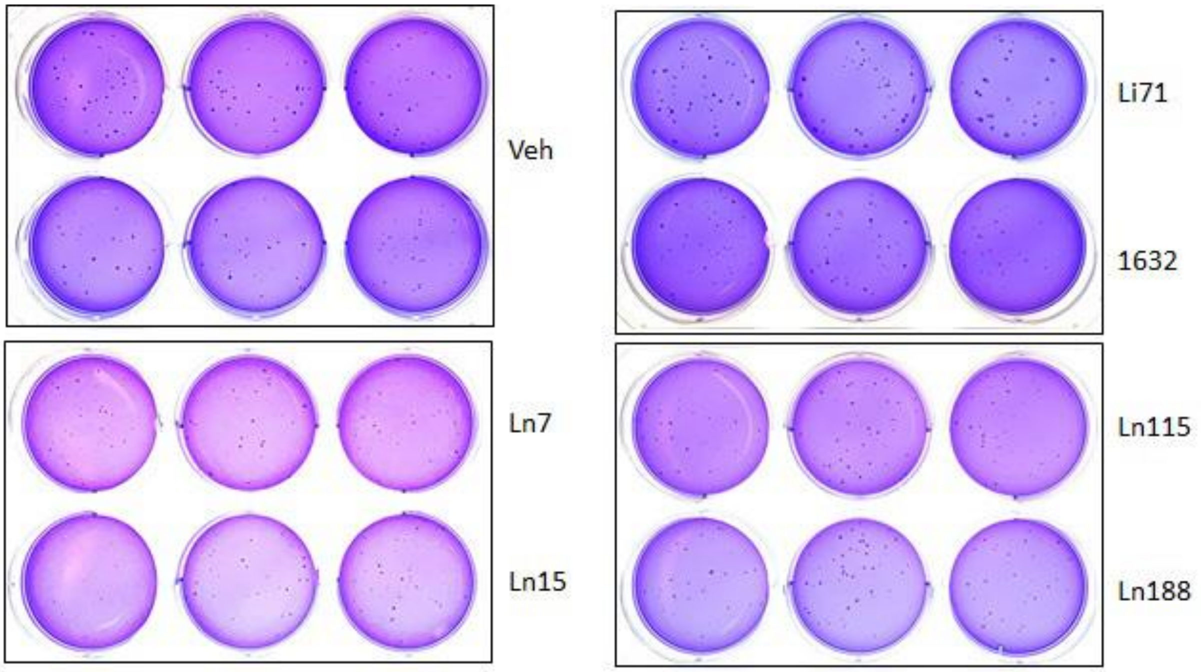

(B)

Figure S4. A. Colony formation assays: DUNE cells were seeded in 500 cells/well. Chemicals were added one day after cell seeding (n=3 wells/chemical). Medium with chemical at the concentration of 60uM was changed every four days for 14 days. Colonies were stained with

0.005% crystal violet staining for 2 hours. ImageJ was used to analyze colony size and numbers (circularity was set at 0.5 -1, which is equivalent to 50uM in size). Only colonies larger than 50uM were countered. **B. Colony formation assays:** DUNE(KO) cells were seeded in 500 cells/well. Chemicals were added one day after cell seeding (n=3 wells/chemical). Medium with chemical at the concentration of 60uM was changed every four days for 14 days. Colonies were stained with 0.005% crystal violet staining for 2 hours. ImageJ was used to analyze colony size and numbers (circularity was set at 0.5 -1, which is equivalent to 50uM in size). Only colonies larger than 50uM were countered.

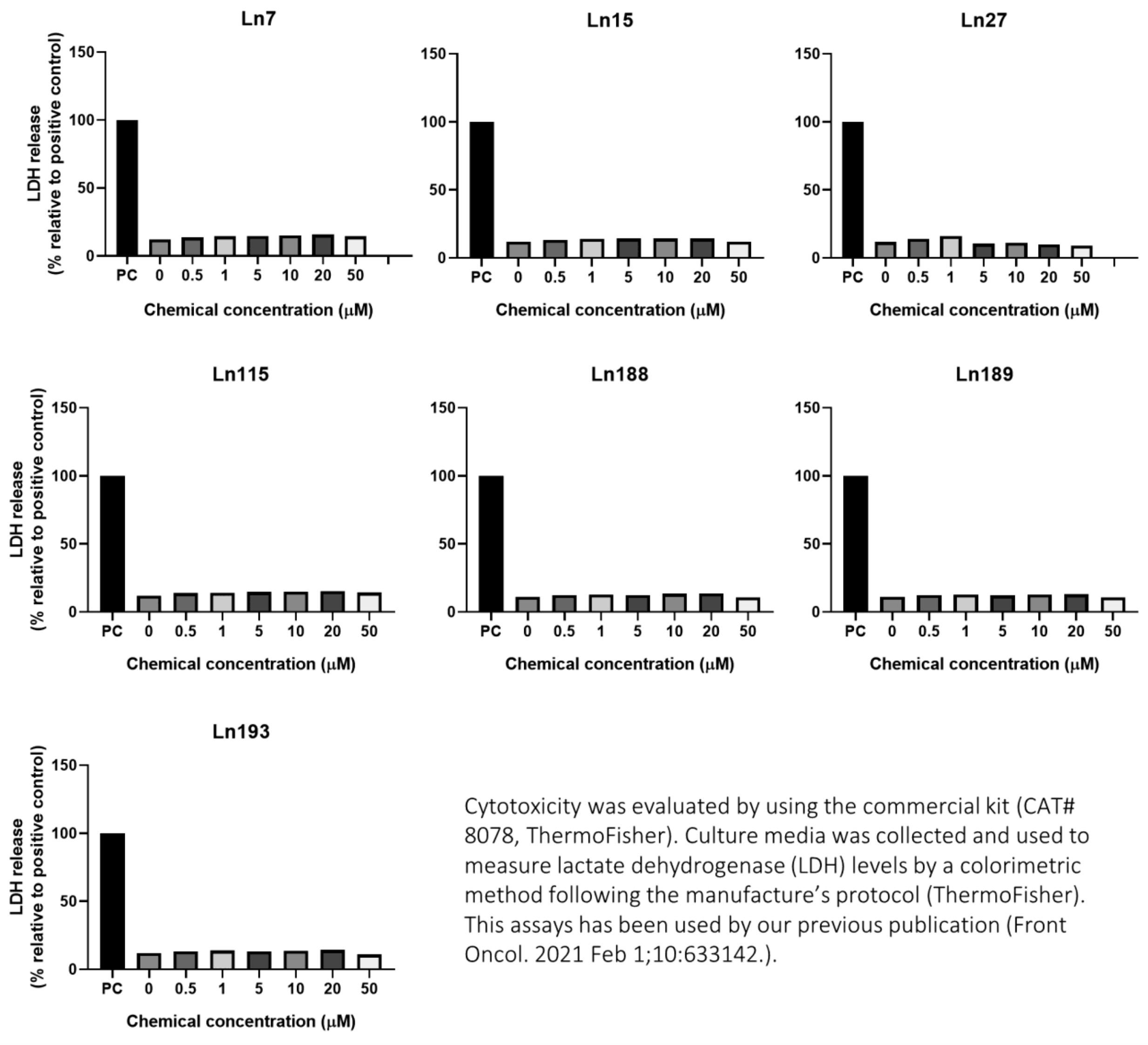

Cytotoxicity was evaluated by using the commercial kit (CAT# 8078, ThermoFisher). Culture media was collected and used to measure lactate dehydrogenase (LDH) levels by a colorimetric method following the manufacture's protocol (ThermoFisher). This assays has been used by our previous publication (Front Oncol. 2021 Feb 1;10:633142.).

Figure S5. Cytotoxicity to cells by measuring lactate dehydrogenase (LDH) release.

Table S1. Real time-qPCR Primer Information

Primer Name	Forward Primer Sequence (5'-3')	Reverse Primer Sequence (5'-3')
ALDH1A2	TTGCAGGGCGTCATCAAAAC	ACACTCCAATGGGTTCATGTC
ASCL1	CCCAAGCAAGTCAAGCGACA	AAGCCGCTGAAGTTGAGCC
CHGB	CGAGGGGAAGATAGCAGTGAA	CAGCATGTGTTTCCGATCTGG
FOXC1	TGTTCGAGTCACAGAGGATCG	ACAGTCGTAGACGAAAGCTCC
FOXD3	TCACGCACCAATTCTAACGC	CACGGCTTGCTTACTGAAGG
GAPDH	GGACCTGACCTGCCGTCTAGAA	GGTGTCGCTGTTGAAGTCAGAG
HEY1	GTTCGGCTCTAGGTTCCATGT	CGTCGGCGCTTCTCAATTATTC
HMGA2	AGTCCCTCTAAAGCAGCTCAAAAG	GCCATTTCCTAGGTCTGCCTC
ID4	GGCCACTCAAGCAGCATTTG	TCTGGTTGCCTGGTTAGGAC
LIN28B	TGTAGTCTACCTCCTCAGCCAA	ATTCTGCTTCCTGTCTTCCCTG
miR-let-7d	CCAGCTGGGAGAGGTAGTAGGTTGC	CTGGTGTCGTGGAGTCGGCAATT
SCGN	GGCCATTTCTGAGGCTAAACT	GGGCTCCTGTTTTACTAACATCA
SIX2	AAGGCACACTACATCGAGGC	CACGCTGCGACTCTTTTCC
SOX2	GCCGAGTGGAAACTTTTGTCG	GGCAGCGTGTACTTATCCTTCT
SYP	TTAGTTGGGGACTACTCCTCG	GGCCCTTTGTTATTCTCTCGGTA
SYT4	ATGGGATACCCTACACCCAAAT	TCCCGAGAGAGGAATTAGAACTT
U6	GCTTCGGCAGCACATATACTAAAAT	CGCTTCACGAATTTGCGTGTCAT
**Primer Name**	**Stem Loop Primer Sequence (5'-3')**	
miR-let-7d	CTCAACTGGTGTCGTGGAGTCGGCAATTCAGTTGAGAACTATGC	
U6	CTCAACTGGTGTCGTGGAGTCGGCAATTCAGTTGAGAAAAATATG	

Table S2. Primary Antibodies List

Antibody	Vendor	Catalogue Number	Application	Dilution
CD44	eBioscience	14-0441-82	WB, FC, IF	1:500
CD133	eBioscience	14-1331-82	WB, FC, IF	1:500
β-Actin	Santa Cruz	sc-1615	WB, IF	1:1000
SOX2	Cell signal	3579	WB	1:1000
HMGA2	ThermoFisher	PA5-21320	WB	1:1000
SYP	Santa Cruz	Sc-17750	WB, IF	1:1000

*FC = Flow Cytometry

*IF = Immunofluorescence

*WB = Western Blot

Table S3. CD133 and CD44 positive cell populations in the FACS studies in figure 7 were listed.

	% of CD133+ cells	% CD44+ cells
DUNE	46.1 ± 5.6	76.6 ± 9.5
DUNE(KO)	15.1 ± 1.3	12.2 ± 4.2
DUNE(veh)	60.8 ± 6.1	84.7 ± 4.1
DUNE(Ln7)	37.1 ± 4.2	61.6 ± 3.2
DUNE(veh)	65.4 ± 6.8	91.1 ± 5.3
DUNE(Ln15)	14.7 ± 2.1	70.1 ± 4.6
DUNE(veh)	62.6 ± 8.6	94.9 ± 1.3
DUNE(Ln115)	30 ± 4.5	54.3 ± 6.0
